# Regulation of S100As Expression by Inflammatory Cytokines in Chronic Lymphocytic Leukemia

**DOI:** 10.3390/ijms23136952

**Published:** 2022-06-22

**Authors:** Olivera Mitrović Ajtić, Tijana Subotički, Miloš Diklić, Dragoslava Đikić, Milica Vukotić, Teodora Dragojević, Emilija Živković, Darko Antić, Vladan Čokić

**Affiliations:** 1 Institute for Medical Research, National Institute of Republic of Serbia, University of Belgrade, 11129 Belgrade, Serbia; tijana@imi.bg.ac.rs (T.S.); milos.diklic@imi.bg.ac.rs (M.D.); dragoslava@imi.bg.ac.rs (D.Đ.); milica.tosic@imi.bg.ac.rs (M.V.); teodora.dragojevic@imi.bg.ac.rs (T.D.); ema.zivkovic55@gmail.com (E.Ž.); vl@imi.bg.ac.rs (V.Č.); 2 Lymphoma Center, Clinic for Hematology, University Clinical Center of Serbia, 11000 Belgrade, Serbia; darko.antic1510976@gmail.com; 3 Faculty of Medicine, University of Belgrade, 11000 Belgrade, Serbia

**Keywords:** inflammation, IL-6, IL-10, CLL, prognostic markers

## Abstract

The calcium-binding proteins S100A4, S100A8, and S100A9 are upregulated in chronic lymphocytic leukemia (CLL), while the S100A9 promotes NF-κB activity during disease progression. The S100-protein family has been involved in several malignancies as mediators of inflammation and proliferation. The hypothesis of our study is that S100A proteins are mediators in signaling pathways associated with inflammation-induced proliferation, such as NF-κB, PI3K/AKT, and JAK/STAT. The mononuclear cells (MNCs) of CLL were treated with proinflammatory IL-6, anti-inflammatory IL-10 cytokines, inhibitors of JAK1/2, NF-κB, and PI3K signaling pathways, to evaluate S100A4, S100A8, S100A9, and S100A12 expression as well as NF-κB activation by qRT-PCR, immunocytochemistry, and immunoblotting. The quantity of S100A4, S100A8, and S100A9 positive cells (*p* < 0.05) and their protein expression (*p* < 0.01) were significantly decreased in MNCs of CLL patients compared to healthy controls. The S100A levels were generally increased in CD19^+^ cells compared to MNCs of CLL. The S100A4 gene expression was significantly stimulated (*p* < 0.05) by the inhibition of the PI3K/AKT signaling pathway in MNCs. IL-6 stimulated S100A4 and S100A8 protein expression, prevented by the NF-κB and JAK1/2 inhibitors. In contrast, IL-10 reduced S100A8, S100A9, and S100A12 protein expressions in MNCs of CLL. Moreover, IL-10 inhibited activation of NF-κB signaling (4-fold, *p* < 0.05). In conclusion, inflammation stimulated the S100A protein expression mediated via the proliferation-related signaling and balanced by the cytokines in CLL.

## 1. Introduction

Chronic lymphocytic leukemia (CLL) is the most common malignancy in the elderly population characterized by increased proliferation and accumulation of B monocytes in the bone marrow [1]. CLL cells are usually long-lived, while rapidly undergoing spontaneous apoptosis in vitro [1,2], which means that the microenvironment and its factors play a significant role in preventing the apoptosis of tumor cells [1,3]. Previous results showed that interleukins, including IL-4, IL-6, and IL-10 are increased in the plasma and tissue of patients with CLL and they play a significant role in preventing apoptosis of these cells [4,5,6]. Despite the emergence of new treatment strategies, there is an increased need for more specific forms of therapy, particularly in relation to the modulation of inflammatory markers [4,6].

S100As proteins belong to a group of low-molecular-weight proteins with a significant role in the regulation of inflammation-related processes in a large number of malignancies including CLL [7,8]. In addition, previous research shows that S100As proteins play a significant role in predicting the therapeutic response of patients susceptible to inflammation including patients with CLL [9]. This creates a potential approach to overcoming drug resistance in multiple cancers, including CLL. The S100A9 gene expression and immunopositive cells are increased in progressive CLL in correlation with the stimulation of the nuclear factor kappa B (NF-κB) signaling pathway [10]. We previously reported that heterodimeric S100A8/9 inhibits ERK1/2 signaling mediated by Toll-like receptor 4 (TLR4) in myeloproliferative neoplasms [11]. TLR4 expression was decreased in mononuclear cells (MNCs) of CLL patients [12,13]. These suggest potentially increased levels of factor S100A8/9, as a ligand for TLR4, through negative feedback regulation, while S100A8/A9 also increases IL-6 secretion in cultured microglial cells [14].

A previous study showed that elevated IL-6 levels in plasma were in positive correlation with a poorer medical outcome in CLL patients [15]. Increased plasma IL-6 levels have also been reported in various nonmalignant diseases [16]. Patients with CLL have increased plasma levels of anti-inflammatory IL-10 [17]. Anti-inflammatory marker IL-10 plays the opposite role to proinflammatory markers and reduces their expression in the plasma of patients with CLL [18]. The NF-κB signaling pathway activated in CLL patients stimulates IL-6 production and secretion which indirectly stimulates the JAK2/STAT3 signaling pathway activation [19]. The blockade of IL-6 or its receptor may improve CLL resistance to the treatment. IL-6 and JAK2/STAT3 act as feedback that stimulates the process of tumorigenesis and metastasis in some types of malignancies [20].

Different types of cancer cells increasingly produce and secrete IL-6 because of the constant stimulation of STAT3 or NF-κB [21]. Additionally, secreted IL-6 keeps STAT3 and NF-κB signaling pathways activated in direct or indirect ways [22,23]. Once activated, both transcription factors regulate the gene expression involved in survival, proliferation, and immunosuppression in cancer cells [23].

The hypothesis of our study was that inflammation induces proliferation and apoptosis related to signaling pathways in CLL. We targeted inflammatory S100A proteins and their regulation by inflammatory cytokines mediated by JAK2-STAT, PI3K-AKT, and NF-κB signaling pathways in patients with CLL.

## 2. Results

### 2.1. The Quantity of S100As Immunopositive MNCs and CD19^+^ Cells in CLL

**Figure 1:**
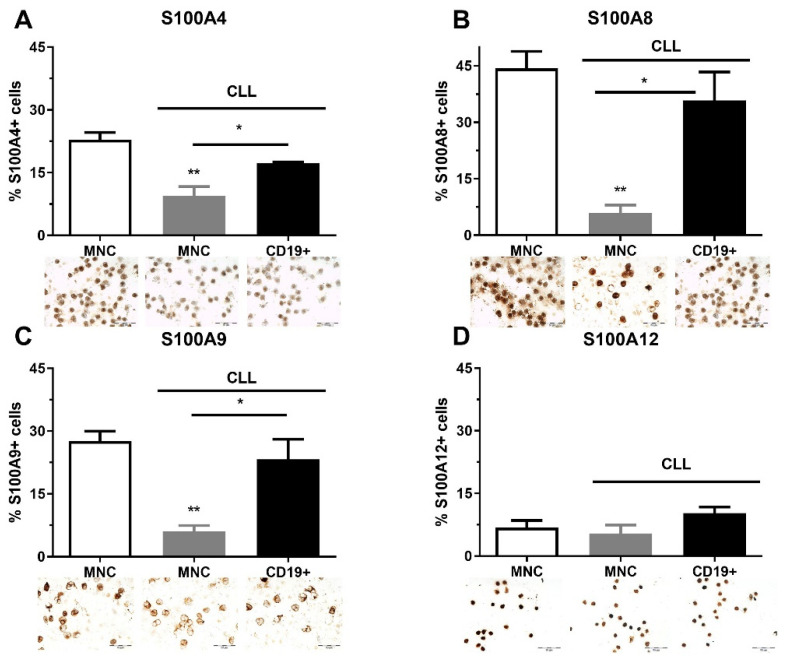
Level of pro-inflammatory S100As positive cells in mononuclear cells (MNCs) and CD19^+^ cells of peripheral blood of chronic lymphocytic leukemia (CLL). The number of (**A**) S100A4, (**B**) S100A8, (**C**) S100A9, and (**D**) S100A12 cells in MNCs and CD19^+^ cells of CLL. Data results are presented as mean ± SD (*n* = 3–4). * *p* < 0.05, ** *p* < 0.01 versus MNCs from healthy donors.

As part of clinical analyses, FISH analysis was performed on CLL patients at diagnosis. Although this analysis was not performed on 58.3% of patients, a large number of patients had deletions (21.7%, Del 13q, 11q, 17p), and Trisomy 12 (10%) (Table S1, see Supplementary Materials). We also found that the immunoglobulin heavy chain gene (IgHV) was mutated in nine CLL patients and was unmutated in four of them (Table S1). We analyzed the quantity of S100A4, S100A8, S100A9, and S100A12 immunopositive MNCs and CD19^+^ cells in CLL patients regardless of genetic abnormalities. S100A4, S100A8, and S100A9 had a similar pattern of expression and showed a higher level of immunopositive cells in the MNCs of healthy controls than in CLL patients, and demonstrated a higher level of expression in CD19^+^ cells than in MNCs of CLL patients (Figure 1). The percentage of S1000A8 (*p* < 0.01, Figure 1B) and S100A9 positive cells (*p* < 0.01, Figure 1C) were significantly decreased in MNCs of CLL patients in comparison to healthy controls. Generally, the examined S100A proteins were decreased in MNCs of CLL patients.

### 2.2. S100As Protein Expression in MNCs and CD19^+^ Cells of CLL Patients

**Figure 2:**
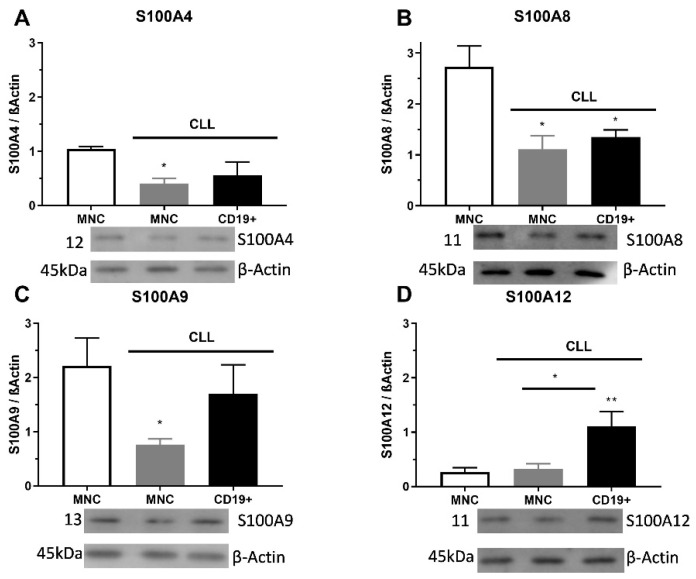
Level of pro-inflammatory S100As proteins in mononuclear cells (MNCs) and CD19^+^ cells of peripheral blood of chronic lymphocytic leukemia (CLL). (**A**) S100A4, (**B**) S100A8, (**C**) S100A9, and (**D**) S100A12 protein levels in MNCs and CD19^+^ cells of CLL were determined by Western blotting. Data results are presented as mean ± SD (*n* = 4). * *p* < 0.05, ** *p* < 0.01 versus MNCs from healthy donors.

The S100A4 level of protein was significantly decreased in MNCs of CLL than in control samples (*p* < 0.05, Figure 2A). The S100A8 protein expression had a significantly lower level of appearance in MNCs and CD19^+^ cells than in samples of control cells (*p* < 0.05, Figure 2B). The S100A9 protein expression had significantly lower expression levels in MNCs (*p* < 0.05) than in healthy control cells (Figure 2C). Significant differences were detected between the expression levels of S100A12 protein in MNCs and CD19^+^ cells, in favor of CD19^+^ cells (*p* < 0.01, Figure 2D). Additionally, S100A4 immunopositive cells showed a pattern of individual positive cells, while S100A8 and S100A9 had a trend of cluster positive cells. Similar to immunopositive cells, the S100As protein expression was reduced in MNCs of patients with CLL.

### 2.3. S100As Gene Expression in MNCs of CLL after IL-6 and IL-10 Treatment

**Figure 3:**
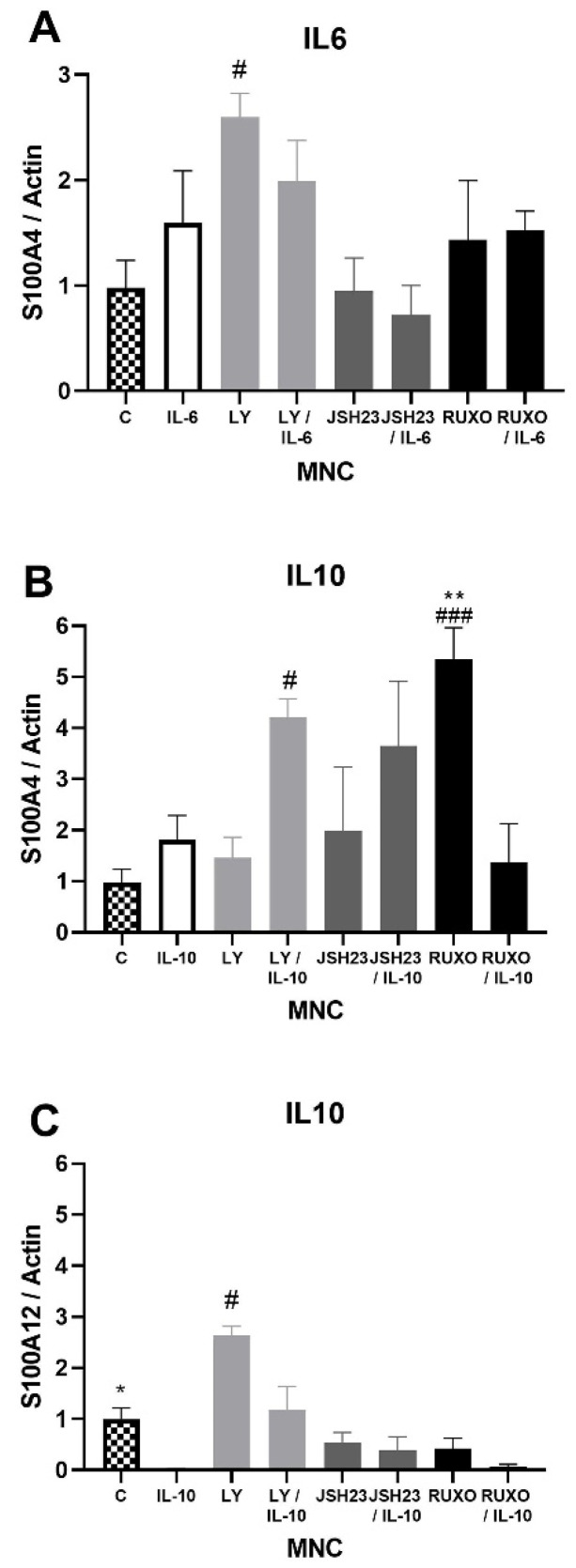
Inflammatory interleukin-6 (IL-6) and IL-10 induction of the S100As gene expression in mononuclear cells (MNCs) of peripheral blood of chronic lymphocytic leukemia (CLL). MNCs of CLL were treated 1 h by 20 µg/mL IL-6 in combination with and without 0.6 nM Ruxolitinib, 5 nM LY294002, and 0.75 nM NF-κB inhibitor JSH23 and levels of (**A**) S100A4; or 30 µg/mL IL-10 in combination with and without 1.2 nM Ruxolitinib, 10 nM LY294002, and 1.5 nM NF-κB inhibitor JSH23 and levels (**B**) S100A4 and (**C**) S100A12 were defined. Data results are presented as mean ± SD (*n* = 5). # *p* < 0.05, ### *p* < 0.001 versus MNCs (control), * *p* < 0.05, ** *p* < 0.01 versus IL-6 or IL-10 treated MNC.

S100A4 gene expression was significantly stimulated after treatment with an inhibitor of the PI3K signaling pathway (Ly294002) in comparison to nontreated MNCs of CLL patients (*p* < 0.05, Figure 3A). Both IL-6 and IL-10 increased S100A4 gene expression, but those differences were not statistically significant (Figure 3A,B). Ly294002 in combination with IL-10 significantly stimulated the level of S100A4 expression (*p* < 0.05, Figure 3B). The inhibitor of the JAK/STAT signaling pathway Ruxolitinib also significantly stimulated S100A4 gene expression (*p* < 0.001, Figure 3B). IL-10 significantly reduced the level of S100A12 gene expression in MNCs of CLL in comparison to nontreated MNCs (*p* < 0.05), while Ly294002 significantly stimulated the level of S100A12 gene expression (*p* < 0.05, Figure 3C). IL-6 and IL-10 did not significantly change the S100A8 and S100A9 gene expression (not shown). The S100As gene expression was generally mediated by the PI3K signaling pathway, while JAK/STAT signaling is included in the regulation of the S100A4 gene expression.

### 2.4. S100As Protein Expression after Treatment of MNCs with the Proinflammatory Cytokine IL-6

**Figure 4:**
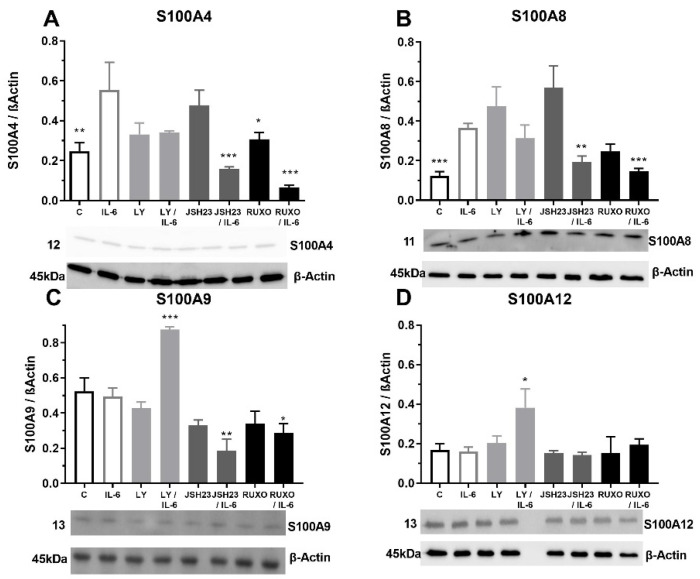
Proinflammatory IL-6 increase of S100As protein expression in mononuclear cells (MNCs) of peripheral blood of chronic lymphocytic leukemia (CLL). MNCs of CLL patients were treated 1 h by 20 µg/mL IL-6 in combination with and without 0.6 nM Ruxolitinib, 5 nM LY294002 and 0.75 nM NF-κB inhibitor JSH23 and levels of (**A**) S100A4, (**B**) S100A8, (**C**) S100A9, and (**D**) S100A12 were defined. Data results are presented as mean ± SD (*n* = 5). * *p* < 0.05, ** *p* < 0.01, *** *p* < 0.001 versus IL-6 treated MNC.

The MNCs of CLL patients were exposed to pro-inflammatory cytokine IL-6 to examine the S100As protein levels. Our results showed that IL-6 significantly stimulated the level of S100A4 protein expression (*p* < 0.01) in comparison to nontreated MNCs (Figure 4A). Inhibitor of the NF-κB signaling pathway (JSH23) prevented the IL-6 induction of S100A4 protein expression in MNCs (*p* < 0.001) in combination with IL-6 (Figure 4A). The same prevention applies to the inhibitor of the JAK2/STAT3 signaling pathway, Ruxolitinib (*p* < 0.001, Figure 4A). Our results showed that IL-6 significantly increased the level of the S100A8 protein expression in the MNCs of CLL (*p* < 0.001, Figure 4B). Again, JSH23 (*p* < 0.01) and Ruxolitinib (*p* < 0.001) prevented the IL-6 induction of the S100A4 protein expression in MNCs (Figure 4B). IL-6 did not change S100A9 and S100A12 protein expressions in the MNCs of CLL (Figure 4C,D). IL-6 together with JSH23 (*p* < 0.01) and Ruxolitinib (*p* < 0.05) reduced the S100A9 protein expression (Figure 4C). In contrast, IL6 together with PI3K inhibitor (Ly294002) significantly increased S100A9 (*p* < 0.001) and S100A12 (*p* < 0.05) protein expressions (Figure 4C,D). IL-6 induction of the S100A proteins is mediated via NF-κB and JAK2/STAT3 signaling pathways.

### 2.5. S100As Protein Expression after Treatment of MNCs with Anti-Inflammatory Cytokine IL-10

**Figure 5:**
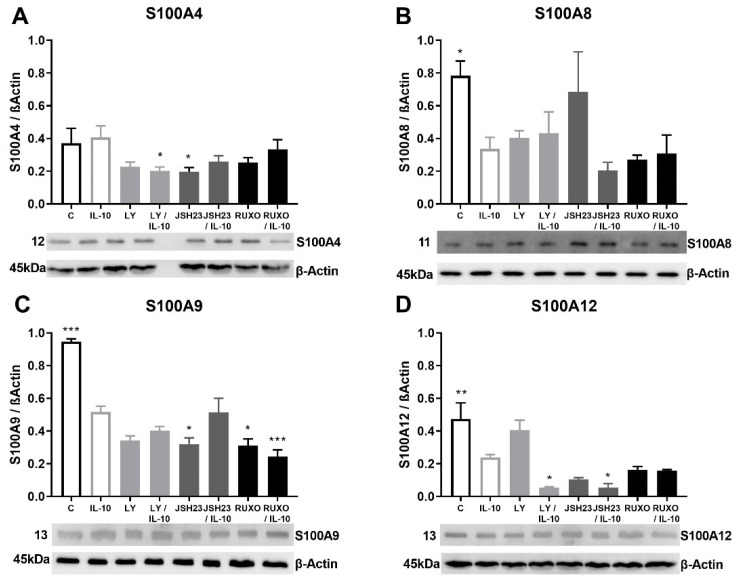
Anti-inflammatory interleukin-10 (IL-10) induction of S100As protein expression in mononuclear cells (MNCs) of peripheral blood of chronic lymphocytic leukemia (CLL). MNCs of CLL were treated 1 h by 30 µg/mL IL-10 in combination with and without 1.2 nM Ruxolitinib, 10 nM LY294002, and 1.5 nM NF-κB inhibitor JSH23 and levels of (**A**) S100A4, (**B**) S100A8, (**C**) S100A9, and (**D**) S100A12 were defined. Data results are presented as mean ± SD (*n* = 5). * *p* < 0.05, ** *p* < 0.01, *** *p* < 0.001 versus IL-10 treated MNC.

Anti-inflammatory cytokine IL-10 generally reduced the S100As (except S100A4) protein expressions in MNCs of CLL patients (Figure 5). The S100A4 protein expression was significantly decreased mutually by the PI3K inhibitor (Ly294002) and IL-10 (*p* < 0.05) in MNCs of CLL (Figure 5A). IL-10 significantly reduced level of S100A8 (*p* < 0.05), S100A9 (*p* < 0.001), and S100A12 (*p* < 0.01) protein expressions in MNCs of CLL (Figure 5B–D). The most significant decrease in S100A9 protein expression was detected after mutual treatment of MNCs with Ruxolitinib and IL-10 (*p* < 0.001, Figure 4C). S100A12 protein expression was additionally decreased after combined treatment of Ly294002 and IL-10, as well as the combination of JSH23 and IL-10 (*p* < 0.05, Figure 4D). IL-10 stimulated reduction of the S100As protein levels was magnified by inflammation-related signaling pathways in CLL.

### 2.6. pNF-κB/NF-κB Signaling in CD19^+^ and MNCs of CLL after Treatment with IL-6 and IL-10

**Figure 6:**
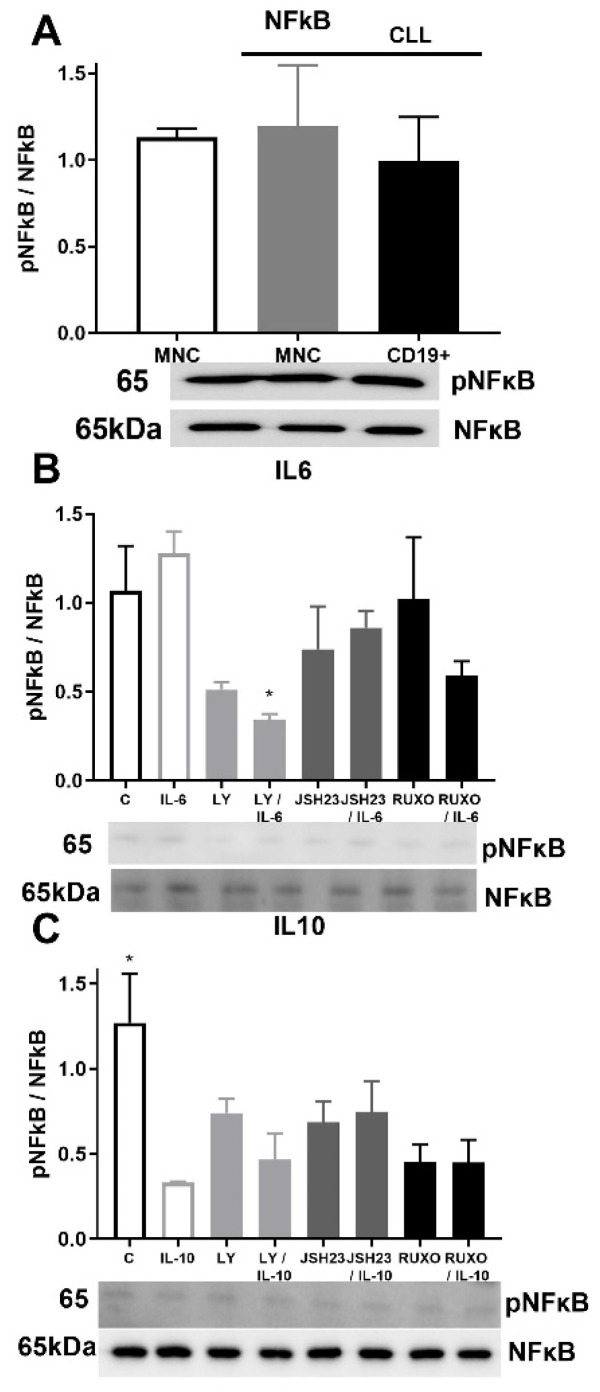
Activation of inflammatory NF-κB signaling pathway in mononuclear cells (MNCs) of peripheral blood of chronic lymphocytic leukemia (CLL). Activation of NF-κB signaling in (**A**) MNCs and CD19^+^ cells of CLL (*n* = 3); (**B**) MNCs treated 1 h by 20 µg/mL IL-6 with and without 0.6 nM Ruxolitinib, 5 nM LY294002, and 0.75 nM JSH23; (**C**) MNCs treated 1 h by 30 µg/mL IL-10 with and without 1.2 nM Ruxolitinib, 10 nM PI3K inhibitor, and 1.5 nM NF-κB inhibitor JSH23. Data are presented as mean ± SD (*n* = 5). * *p* < 0.05, versus IL-6 or IL-10 treated MNCs.

Activation of NF-κB signaling was similar in MNCs of CLL and healthy donors as well as in CD19^+^ cells of CLL (Figure 6A). Phosphorylation of NF-κB was reduced after joined treatment of the PI3K inhibitor (Ly294002) and IL-6 (*p* < 0.05, Figure 6B). Activation of NF-κB signaling was significantly reduced by IL-10 in MNCs of CLL compared to nontreated MNCs (*p* < 0.05, Figure 6C). Anti-inflammatory cytokine IL-10 demonstrated an influence on NF-κB signaling with inhibition of the activation.

## 3. Discussion

A previous study has shown that S100A12, S100A8, and S100A9 are a subgroup of S100 proteins that are usually increased in serum samples from patients with a different type of inflammation [24]. In several types of cancer cells, S100A4 is responsible for their ability to form metastases, promoting their invasiveness [25]. Similarly, S100A8 and S100A9 are also linked to anticancer properties, tumor progression, and the development of tumor metastasis [26]. S100A12 is constitutively expressed in neutrophils while it is induced by lipopolysaccharide (LPS) and tumor necrosis factor-alpha (TNF-α) in peripheral blood monocytes and by IL-6 in human macrophages [27,28]. IL-6 via JAK/STAT3 signaling has mediated S100A8/A9 induction [29]. The JAK1/2 inhibitor Ruxolitinib has restored sensitivity to the B-cell chronic lymphocytic leukemia/lymphoma 2 (BCL2) inhibitor venetoclax in AML patient cells [30]. Overexpression of the antiapoptotic protein BCL2 is a hallmark of CLL [31]. Venetoclax selective targeting of BCL2 induced substantial responses (71–79%) in patients with relapsed CLL, while complete remissions occurred in 20% of the patients [32]. The degradation of BCL2 was blocked by S100A8 and S100A9 stimulation [33]. The presented results showed that the proinflammatory marker IL-6 stimulates the expression of S100A4 and S100A8 proteins in CLL, mediated by JAK1/2- and NF-κB-signaling. These findings support the inflammation’s role in the overexpression of antiapoptotic protein BCL2 and CLL progression.

NF-κB is constitutively activated in peripheral blood low-density cells of patients with CLL [34]. Our results showed that IL-6 in combination with the PI3K inhibitor significantly reduced NF-κB phosphorylation, while IL-10 also inhibited the NF-κB signaling in MNCs of CLL. Numerous studies suggest that NF-κB should be studied as a potential place for the synthesis of specific therapy in CLL [35,36]. S100A9 is an activator of the NF-κB pathway during CLL progression [37]. According to the results of our study, the IL-6 induction of the S100As proteins is regulated via the NF-κB and JAK2/STAT3 signaling pathways. These observations support the involvement of proliferation-related signaling in the inflammatory response of CLL cancer cells with the ability of sustained proliferation.

It has been reported that high levels of AKT phosphorylation occur in high-risk patients with CLL, while upregulated S100A4 and S100A9 gene expression has been described in an Akt-induced CLL mouse model [38]. S100A4 is regulated by PI3K/AKT signaling in cancers [39]. In our study, IL-6 had no effects on S100A9 and S100 A12 gene expression, but with inhibition of PI3K signaling, the S100As proteins were highly upregulated. The opposite effects were observed by the inhibition of PI3K signaling during IL-10 treatment of MNCs. The phosphorylated PI3K/AKT signaling pathway can be considered a new biomarker of severe clinical outcomes in CLL patients, and the inhibition of those signaling pathways may be a possible treatment action option for these patients.

Idelalisib is an inhibitor of PI3Kδ that has been used since 2014 in the treatment of patients with severe clinical outcomes together with rituximab [40]. In some randomized studies idelalisib plus rituximab as opposed to placebo plus rituximab in patients with relapsed CLL showed that idelalisib improved progression-free survival and overall survival compared with rituximab alone [41]. The PI3K signaling pathway is activated in CLL patient’s cells, while hyperactivation of PI3K/AKT-signaling induces acute cell death in CLL cells [41].

The precision medicine approach in CLL patient’s treatment includes both chemoimmunotherapy and biological drugs that depend on mutations in TP53 and IGHV genes [42]. Previous genetic studies have shown that the NF-kB signaling pathway is constitutively activated in patients with CLL and disrupts the control of apoptosis and proliferation of tumor cells, causing their increased proliferation [32]. According to our results, IL-10 inhibited phosphorylation of NF-κB in MNCs of CLL which could also have an impact on precision medicine of CLL.

A previous study has shown that increased IL-10 levels lead to progressive immunosuppression in CLL patients [17]. It has been demonstrated that IL10 agonists may support the current therapeutic treatment for CLL by potentiating the host’s anticancer cell response [17]. In our study IL-10 reduced the expression of S100A8, S100A9, and S100A12 proteins in CLL, attributing their prognostic potential to therapy of CLL.

According to our results, the examined S100As proteins are generally reduced in MNCs of CLL. IL-6 stimulation was JAK1/2- and NF-κB-dependent for S100A8 and S100A9 proteins, in contrast to the anti-inflammatory cytokine IL-10. In addition, IL-10 inhibited the NF-κB PI3K signaling pathway. The levels of the S100As, especially S100A8 can be diagnostic predictive markers for CLL. Future studies should focus on the S100A effects on proliferation signaling and cell cycle in CLL cancer cells during treatment with current and novel therapeutic agents.

## 4. Materials and Methods

In our study, we included 60 CLL patients’ samples. All patients’ samples were collected at the time of diagnosis and before treatment. All 60 CLL patients included in our study signed the consent form approved by a local ethical committee in accordance with the Declaration of Helsinki. We collected 30 mL of peripheral blood from all subjects, and it was drawn and collected in disodium EDTA. MNCs, after the process of isolation, were washed in PBS and resuspended in an RPMI-1640 medium (Biowest, Nuaillé, France). After that, MNCs were pretreated for one hour with 1 μM or 1.2 μM Ruxolitinib (RUXO, JAK1/2 inhibitor, Cayman Chemical Company, Ann Arbor, MI, USA), 5 μM or 10 μM Ly294002 (PI3K inhibitor, Cell Signalling Technology, Inc, Danvers, MA, USA), and 0.75 μM or 1.5 μM JSH23 (NF-κB inhibitor, Sigma-Aldrich, Darmstadt, Germany), and treated for one hour with IL-6 or IL-10 (20 ng/mL and 30 ng/mL, Miltenyi Biotec, Bergisch Gladbach, Germany). Then MNCs were washed in PBS and treated with RIPA lysis buffer at 4 °C for 30 min. After those treatments, MNCs were centrifuged at 10000× *g* at 4 °C for 15 min. B-lymphocyte antigen CD19^+^ cells were purified using MACS cell separation and CD19^+^ microbeads from the peripheral blood of patients with CLL, according to the manufacturer’s protocol (Miltenyi Biotec).

### 4.1. Western Blot Analysis

Isolation of proteins from MNCs of CLL was conducted as previously reported [43] and 30 μg of proteins were tracked on gels and transferred to polyvinylidene difluoride membranes. After this process, the membranes were blocked in 4% milk solution (Serva Electrophoresis GmbH, Heidelberg, Germany) for 1 h at +40 °C. Then, the membranes were incubated with appropriate primary antibodies. The primary antibodies used in our study were against S100A4 (Cell Signaling Technology, Inc.), S100A8 (Abcam, Cambridge, UK), S100A9 (Abcam), S100A12 (Elabscience Biotechnology Co., Ltd, Wuhan, China), β-actin (R&D Systems, Inc, Minneapolis, MN, USA), phospho-NF-κB p65 (Ser536, Cell Signaling Technology), and NF-κB (Santa Cruz Biotechnology, Dallas, TX, USA). Goat antirabbit immunoglobulin (R&D Systems) was used as a secondary antibody, except for β-actin where goat antimouse immunoglobulin was used. The degree of expression of the tested proteins was determined by densitometric analysis and the ChemiDoc Imaging System (Bio-Rad Laboratories, Hercules, CA, USA). The obtained values were normalized to β-actin.

### 4.2. Immunocytochemistry Analysis

Immunocytochemical staining was performed on cytospin mononuclear samples isolated from 10 healthy controls and 30 CCL patients. Mononuclear cells (2 × 10^4^ cells per slide) were fixed to super frost plates using methanol. Blocking of nonspecific staining derived from endogenous peroxidase was performed by treating the samples with 3% hydrogen peroxide. The samples were then treated and incubated with appropriate primary antibodies overnight at +40 °C. The streptavidin-biotin technique (LSAB+/HRP Kit, DAKO, Glostrup, Denmark) was used for staining. The DAKO Liquid DAB+ Substrate/Chromogen System (DAKO) was used to visualize the positive immunoreactivity, while the contrast of unstained nuclei was performed using Mayer’s hematoxylin (Merck, Whitehouse Station, NJ, USA). As a negative control, we used samples treated with PBS without a primary antibody. From each section, five fields with the average number of immunoreactive cells were imaged and processed. Imaging was performed using a light microscope (Olympus AX70, Hamburg, Germany), while immunoreactive cell counting was performed using a special program (Analysis Pro 3.1). All images had an 40x magnification.

### 4.3. Isolation of RNA and RT-PCR

RNA was isolated from CLL patients’ mononuclear cells using Trizol. Determination of isolated RNA concentration was performed using an Ultrospec 3300 spectrophotometer (Amersham Pharmacia, Upsala, Sweden). Equal RNA concentrations of all samples were used for transcription into cDNA using the Maxima First Strand cDNA Synthesis kit (Thermo Fisher Scientific, Waltham, MA, USA).

### 4.4. RT-q PCR Analysis

Real-time quantitative PCR analysis for S100A4, S100A8, S100A9, and S100A12 were performed using appropriate primers and Maxima SYBR Green/ROX qPCR master mix (Thermo Scientific, Cambridge, UK) at Mastercycler EP RealPlex (Eppendorf AG, Hamburg, Germany).

### 4.5. Statistical Analysis

The obtained experimental results are presented as mean ± standard deviation. The statistical program Prism 4 was used for the analysis, while one-way ANOVA and Dunnett’s posttest were used as statistical tests.

## Data Availability

Not applicable.

## Supplementary Materials

The following supporting information can be downloaded at https://www.mdpi.com/article/10.3390/ijms23136952/s1.
