# Supplementary material for: Regulation of S100As Expression by Inflammatory Cytokines in Chronic Lymphocytic Leukemia
Source: Int J Mol Sci. 2022 Jun 22;23(13):6952. doi: 10.3390/ijms23136952 (PMC9267105; doi:10.3390/ijms23136952)
Supplement: Supplementary file 1 [file ijms-23-06952-s001.zip › ijms-1751237-supplementary.pdf]

Supplemental Table S1. FISH analysis in 60 CLL patients.

FISH analysis	*No. Of patients (%)*	*IGHV*	*No. Of patients (%)*
Del11q Trisomy 12	1 (1.65 %)	Mutated	9 (15 %)
Del11q Del 13q	1 (1.65 %)	Unmutated	4 (6.7 %)
Del 13q	8 (13.4 %)	No data	47 (78.3 %)
Del11q	1 (1.65 %)		
Trisomy 12	5 (8.4 %)		
Del13q Del 17p	2 (3.4 %)		
Normal	6 (10 %)		
Negative	1 (1.65 %)		
No data	35 (58,4 %)		
